# Obesity and Preeclampsia: Common Pathophysiological Mechanisms

**DOI:** 10.3389/fphys.2018.01838

**Published:** 2018-12-19

**Authors:** Patricio Lopez-Jaramillo, Juan Barajas, Sandra M. Rueda-Quijano, Cristina Lopez-Lopez, Camilo Felix

**Affiliations:** ^1^Clinic of Metabolic Syndrome, Prediabetes, and Diabetes, Research Department, FOSCAL, Floridablanca, Colombia; ^2^Masira Institute, Medical School, Universidad de Santander, Bucaramanga, Colombia; ^3^Facultad de Ciencias de la Salud Eugenio Espejo, Universidad Tecnologica Equinoccial, Quito, Ecuador; ^4^Obstetric Department, FOSCAL, Floridablanca, Colombia

**Keywords:** preeclampsia, obesity, endothelial dysfunction, nitric oxide, cardiovascular risk

## Abstract

Preeclampsia is a disorder specific of the human being that appears after 20 weeks of pregnancy, characterized by new onset of hypertension and proteinuria. Abnormal placentation and reduced placental perfusion associated to impaired trophoblast invasion and alteration in the compliance of uterine spiral arteries are the early pathological findings that are present before the clinical manifestations of preeclampsia. Later on, the endothelial and vascular dysfunction responsible of the characteristic vasoconstriction of preeclampsia appear. Different nutritional risk factors such as a maternal deficit in the intake of calcium, protein, vitamins and essential fatty acids, have been shown to play a role in the genesis of preeclampsia, but also an excess of weight gain during pregnancy or a pre-pregnancy state of obesity and overweight, which are associated to hyperinsulinism, insulin resistance and maternal systemic inflammation, are proposed as one of the mechanism that conduce to endothelial dysfunction, hypertension, proteinuria, thrombotic responses, multi-organ damage, and high maternal mortality and morbidity. Moreover, it has been demonstrated that pregnant women that suffer preeclampsia will have an increased risk of future cardiovascular disease and related mortality in their later life. In this article we will discuss the results of studies performed in different populations that have shown an interrelationship between obesity and overweight with the presence of preeclampsia. Moreover, we will review some of the common mechanisms that explain this interrelationship, particularly the alterations in the L-arginine/nitric oxide pathway as a crucial mechanism that is common to obesity, preeclampsia and cardiovascular diseases.

## Introduction

Obesity is considered a risk factor for preeclampsia and there are many common mechanisms that link obesity with a higher risk of developing preeclampsia (Spradley et al., 2015). Moreover, preeclampsia, similar to obesity, is associated with an increased risk of future cardiovascular diseases for the mother (Bellamy et al., 2007). Preeclampsia is a specific disease of the human being characterized by hypertension, edema of extremities, and proteinuria occurring after 20 weeks of gestation. It affects many organ systems and leads to high maternal mortality and morbidity worldwide (Lopez-Jaramillo et al., 2009). Hypertensive disorders are amongst the most common disorders that affect pregnant women and are major contributors to maternal deaths. In a systematic review conducted by the World Health Organization (WHO), 16% of maternal deaths in developed countries were attributed to hypertensive disorders, 9% in the regions of Africa and Asia, and as high as 25% in Latin America and the Caribbean (Khan et al., 2006). The WHO-review named hypertensive disorders during pregnancy the leading cause of maternal deaths in industrialized countries, responsible for 16% of maternal deaths. Regionally, hypertensive disorders are responsible of 25,000 maternal deaths in Africa, 22,000 in Asia, 3,800 in Latin America and the Caribbean, and 150 maternal deaths in industrialized countries (Khan et al., 2006). The two disorders most associated with hypertension during pregnancy are eclampsia and preeclampsia. Preeclampsia can be characterized by hypertension, proteinuria, edema of extremities, persistent severe headaches, visual disturbances, sudden onset of swelling of hands and feet, and hyperreflexia, amongst many more. It affects coagulation, the renal, respiratory, and central nervous system and can have detrimental consequences on the placenta and the baby (Duley, 2009). Most maternal hypertensive deaths are attributed to eclampsia as opposed to preeclampsia. Eclampsia occurs when preeclampsia goes untreated and the hypertensive mothers experience seizures. It is estimated that hypertensive disorders complicate 5–10% of all pregnancies and preeclampsia arises in 2–8% of them, although it is difficult to gather accurate data on the prevalence of preeclampsia worldwide because of differences in the definitions and in the symptoms that are used as diagnostic criteria (Khan et al., 2006). However, there are important variatons in the prevalence of preeclampsia between lower middle-income countries and high-income countries. For instance, preeclampsia is diagnosed in 3% of all pregnancies in the United States (Wallis et al., 2008), and 3.3% in New Zealand (Stone et al., 1995) while in Colombia it is present in 9% and in Haiti in 17% (Lopez-Jaramillo et al., 2005, 2007). In the United States, from 1987 to 2004 the average annual incidence rate of preeclampsia and gestational hypertension were 2.7 and 2.1% (Wallis et al., 2008). In the same 18-year period, the rate of preeclampsia and gestational hypertension has increased significantly. From 1987–1988 to 2003–2004, the age-adjusted rate of preeclampsia rose by 24.6%. Meanwhile, the rates for gestational hypertension increased by almost threefold from 1.07 to 3.6%. The rate for eclampsia during the same time decreased by 22% from 1.04 in 1987–1995 to 0.82 in 1996–2004, although this change was not significant. The risk of developing preeclampsia is highest amongst women <20 years of age, but women ≥35 years of age also have an increased risk of developing preeclampsia (Wallis et al., 2008).

Although the terminology and methods for classifying and diagnosing the hypertensive disorders of pregnancy differ from country to country and region to region, some common risk factors have been identified. These risk factors include, maternal age, pre-pregnancy overweight and obesity, sedentarism, insulin resistance and diabetes, subclinical infections and inflammation, and nutritional deficiencies during pregnancy as low intake of calcium and essential fatty acids (Lopez-Jaramillo et al., 1989; Lopez-Jaramillo et al., 1990b, 1997, 2005, 2008b, 2011; Lopez-Jaramillo, 1996; Otto et al., 1997, 1999; Herrera et al., 2001, 2005, 2006, 2007; Teran et al., 2001; Catov et al., 2007; García et al., 2007; Sierra-Laguado et al., 2007; Wang et al., 2008; Ramírez-Vélez et al., 2011; World Health Organization [WHO], 2011; Reyes et al., 2012,a,b).

## Obesity, Preeclampsia and Cardiovascular Diseases

The relationship between preeclampsia and obesity has been greatly studied. Similar to how the trend of preeclampsia has increased over the past 25 years, obesity prevalence has also been on the uprise. Over the past 30 years, the percentage of overweight or obese women in the US has increased by 60% (Wang et al., 2008). The WHO estimates that the female prevalence of overweight and obesity is 77% in the United States, 73% in Mexico, 69% in South Africa, 37% in France, 32% in China and 18% in India, with a wide variation within each continent (World Health Organization [WHO], 2011). Numerous studies have shown that obesity is associated with many complications during pregnancy, including fetal overgrowth, fetal malformations, spontaneous miscarriage, gestational diabetes, thromboembolic complications, stillbirth, preterm deliveries, cesarean section, and hypertensive complications (Yogev and Catalano, 2009). A strong direct correlation was found between an increasing body mass index (BMI) and the risk of developing preeclampsia and pregnancy induced hypertension (Fernández Alba et al., 2018). The adjusted risk of developing preeclampsia doubled for overweight mothers with a BMI of 26 kg/m^2^, and almost tripled for obese mothers with a BMI of 30 kg/m^2^ (Bodnar et al., 2005). The increased risk was found to affect not only Caucasian and African American mothers (Bodnar et al., 2007), but also mother from other ethnics around the world (Hauger et al., 2008). Not only were the early or mild forms of preeclampsia found to augment with a raise in the BMI, but also the late and severe forms, which are associated with greater perinatal morbidity and mortality (Catov et al., 2007). Further contributing to the relationship between preeclampsia and obesity, a study found that weight loss reduces the risk of developing preeclampsia (Magdaleno et al., 2012). Despite the fact that weight loss is not recommended during pregnancy, studies have found that excessive maternal weight gain is correlated with an increased risk of preeclampsia (Fortner et al., 2009), thus weight loss is recommended in women with obesity or overweight that are planning to be pregnant (Yogev and Catalano, 2009).

## The Mechanisms Linking Obesity and Preeclampsia

### Metabolic Factors: Hyperinsulinemia and Insulin Resistance in Preeclampsia

Many different mechanisms have been proposed as explanations of the physiopathology of preeclampsia, at a point that it is called the “disease of the theories” (Widmer et al., 2007). Table 1 shows some of the characteristics that are common to obesity and preeclampsia.

**Table 1:** Common characteristics of obesity and preeclampsia.

Characteristic	Obesity	Preeclampsia
Hyperinsulinism	++	++
Insulin resistance	++	++
Leptin	++	++
Adiponectin	--	(+-)?
TNF-α^∗^	++	++
IL-6^∗^	++	++
hs-CRP^∗^	++	++
Lipid profile alterations	++	++
↓ Flow mediated vasodilatation	++	++


*^∗^TNF-α, tumor necrosis factor alpha; IL-6, interleukin 6; hs-CRP, high sensitivity C-reactive protein (CRP).*

The initial phase in the development of preeclampsia is an altered invasion of the cytotrophoblast cells of fetal origin into the uterus and the spiral arterioles, situation that results in a decreased remodeling of these arterioles with a consequent lower blood flow to the placenta (Soma et al., 1982). The placenta in hypoxic conditions releases different substances into the maternal circulation, these include anti-angiogenic soluble fms-like tyrosine kinase 1 (sFlt-1) factors, and pro-inflammatory factors like tumor necrosis factor alpha (TNF-α) (Reyes et al., 2012), which are associated to maternal endothelial dysfunction (Roberts K.A. et al., 2011). As we have demonstrated, these factors are increased in the plasma of preeclamptic women (Teran et al., 2001; Reyes et al., 2012). This sequence of alterations is one of the proposed mechanisms linking obesity to the risk of preeclampsia (Kao et al., 2016), clinical and experimental evidence suggests that obesity may affect placental function and perfusion, through some of the metabolic alterations that are associated to obesity as hyperlipidemia, hyperinsulinemia, or hyperleptinemia; however, the exact mechanisms are not well-known (Hunkapiller et al., 2011). These metabolic markers are known to be elevated in plasma of obese pregnant women and even higher in women with preeclampsia. Moreover, it has been reported that the levels of total serum cholesterol in the first and second trimesters of gestation predict the onset of preeclampsia (Dey et al., 2013), and we have reported a lipid profile alterations consisting of increased levels of low-density lipoproteins (LDLs), low high-density lipoproteins levels (HDLs), and increased levels of triglycerides in women with preeclampsia (Lopez-Jaramillo et al., 1998; Reyes et al., 2012a,b). It has been reported that LDL reduces extravillous cytotrophoblast migration and promotes trophoblast apoptosis (Pavan et al., 2004). Also, high levels of triglycerides and free fatty acids, which are increased in obesity, increase the risk of preeclampsia and are elevated in preeclampsia (Hubel et al., 1996). These two conditions are known to stimulate the nuclear receptor peroxisome proliferator-activated receptor-γ (PPAR-γ). PPAR-γ expression is increased in placentas from preeclamptic pregnancies, and increased levels of this receptor inhibit the invasiveness of trophoblast cells (Fabbrini et al., 2009; Holdsworth-Carson et al., 2010).

One of the most important characteristics of obesity is insulin resistance and hyperinsulinemia, and we have shown that hyperinsulinemia and insulin resistance precede the clinic manifestation of preeclampsia (Sierra-Laguado et al., 2007). Experimental studies showed that hyperinsulinemia produces a shallower implantation site and an intrauterine growth restriction associated with an altered nitric oxide (NO) synthesis (Skarzinski et al., 2009). Moreover, the group of Granger and colleagues, reported that increasing insulin levels toward the end of pregnancy raises blood pressure in rats (Palei et al., 2013). Took together, these clinical and experimental data support the view of the crucial role played by insulin resistance as one of the common mechanisms linking obesity to preeclampsia (López-Jaramillo et al., 2006; Sierra-Laguado et al., 2007).

### Role of Adiponectin, Leptin, and Proinflammatory Cytokines in Preeclampsia

We have determinated the levels of cytokines that are produced in the adipose tissue in patients with diseases as metabolic syndrome and type 2 diabetes mellitus, showing that plasma adiponectin levels are decreased, while proinflammatory cytokines as TNF-α and interleukin-6 (IL-6) are elevated, developing a proinflammatory state characterized by insulin resistance and endothelial dysfunction (Teran et al., 2001; Gómez-Arbeláez et al., 2013; Lopez-Jaramillo et al., 2014; Lopez-Jaramillo, 2016). In patients with severe coronary artery disease, in who we quantify a number of dysmetabolic and inflammatory markers and test of endothelial dysfunction, it was observed that the only differences between patients with and without abdominal obesity was a decrease in plasma concentrations of adiponectin and an increase in leptin plasma levels present in patients with abdominal obesity. Moreover, when we evaluated the vascular reactivity *ex vivo* in rings of the internal mammary artery, we observed a low vascular response to acetylcholine and a high vasoconstriction reaction to angiotensin II (Rueda-Clausen et al., 2010). Leptin and adiponectin are both produced by adipocytes, however, while adiponectin has an anti-inflammatory activity by down-regulating the expression and release of proinflammatory cytokines, leptin has a pro-inflammatory activity. Moreover, adiponectin acts improving the sensibility to insulin but in contrast leptin increases the resistance to the action of insulin. These results suggest that in obese people the increased production of leptin and the decreased production of adiponectin are associated with the systemic low-degree inflammation and insulin resistance, observed in preeclampsia, type 2 diabetes mellitus and cardiovascular diseases, explaining the increased risk of developing these diseases present in people with obesity, particularly abdominal obesity.

In support of this proposal, it has been reported that pregnant obese women that develop preeclampsia have increased leptin levels in relation to healthy pregnant women (Hendler et al., 2005). Also, it has been described that leptin reduces cytotrophoblast proliferation (Liu et al., 2009), and as discussed above, an early alteration observed in preeclampsia is a poor cytotrophoblast proliferation, migration, and invasiveness of these cells into the uterus, it has been suggested that hyperleptinemia may play a role in placental ischemia and the consequent development of preeclampsia (Spradley et al., 2015), as shown by Mendieta Zerón et al. (2012). It was reported that chronic plasmatic leptin elevations in pregnant rats, increased blood pressure and placental factors that have a role in preeclampsia (Palei et al., 2015). Elevations in circulating leptin are associated to increased circulating levels of TNF-α in obese pregnant rats (Palei et al., 2015), and we have shown that women with preeclampsia have increased levels of TNF-α, IL-6 and C-reactive protein (CRP) (Teran et al., 2001). Experimental and clinic reports have demonstrated that TNF-α is increased in response to placental ischemia and hypoxia (Benyo et al., 2001; Peltier et al., 2011). However, other sources must contribute to the increase of circulating levels of TNF-α during preeclampsia, as peripheral blood leukocytes (Chen et al., 1996). Moreover, TNF-α mRNA levels are greater in mononuclear cells from peripheral blood and placental macrophages withdrawn from obese pregnant women (Challier et al., 2008).

We have proposed that the systemic low-degree inflammation that is present in women with preeclampsia is associated to obesity and insulin resistance, and that this low-degree inflammation state is an important mechanism that alters the endothelial function, and that it is proposed as one of the basic mechanism that precede the clinical manifestation of the disease. This proposal is supported by the results of two nested case-control studies that included Colombian pregnant women with preclampsia, demonstrating increased levels of CRP and leukocytes, a state of insulin resistance established by the homeostatic model assessment (HOMA), and a decreased flow-mediated vasodilation, as early as in the first trimester of gestation (García et al., 2007; Sierra-Laguado et al., 2007).

The results of studies that have explored the levels of plasma adiponectin in preeclampsia has been inconsistent, showing increased, decreased or not difference in relation to the observed in healthy pregnancies (Ramsay et al., 2003; Suwaki et al., 2006). Adiponectin activates the endothelial nitric oxide synthase (eNOS); increasing the levels of the vasodilator NO (Zhu et al., 2008) and some cases of preeclampsia course with reduced levels of nitrite, the stable metabolite of NO (Lopez-Jaramillo et al., 2008a). In healthy pregnancies, a positive association between circulating adiponectin concentrations and nitrite levels has been reported, but in preeclampsia there are increased levels of adiponectin with reduced nitrite levels, suggesting that for some reason, not yet determinated, in preeclampsia adiponectin has no act in the eNOS (Eleuterio et al., 2013).

### The Role of the Endothelial L-Arginine-Nitric Oxide Pathway in Obesity and Preeclampsia

Nitric oxide plays an important role in increasing blood flow by relaxing the smooth vascular muscle, it also reduces smooth muscle migration and growth, platelet aggregation and thrombosis, monocyte and macrophage adhesion, and inflammation (Caballero, 2003). Abdominal or central obesity leads to an imbalanced production of fat-derived metabolic products, hormones and adipokines that predispose to a state endothelial dysfunction (Accini et al., 2001). The different mechanisms by which obesity produces endothelial dysfunction have been studied and proven since some years ago by *in vivo* vascular function measurement in peripheral vessels of obese individuals (Toda and Okamura, 2013) showing that obesity reduces endothelium-dependent vasodilatation, eNOS protein expression and endothelial NO production, favoring a thicker media layer, enhancing vasoconstriction and drastically decreasing relaxation. Moreover, as discussed above, proinflammatory adipokines induces endothelial dysfunction. Recently, the Finnish Genetics of Pre-eclampsia Consortium (FINNPEC) cohort have confirmed the women that developed preeclampsia have increased pre-pregnancy BMI and altered inflammatory markers (Jääskeläinen et al., 2018). Leptin, another adipokine that is increased in obesity, induces activation of the NADPH oxidase, which impairs endothelium-dependent vasodilatation by increasing NO degradation (Fortuño et al., 2010). All this leads to a NO deficit which affects endothelial integrity and functioning, that by itself it is deleterious, but in association to pregnancy, where the endothelial function plays a fundamental role in the adequate remodeling of the uterine arteries, and in the hemodynamic adaptations, becomes determinant in the development of preeclampsia-eclampsia, diseases with a high rate of maternal and fetal morbidity and mortality (Witcher, 2018).

During a healthy pregnancy there is an increase of plasma volume, heart rate, cardiac output and on the activity of the renin-angiotensin system (Moutquin et al., 1985), however the blood pressure is lower or similar to the observed in a no pregnancy state, situation that is related with the characteristic increase in the peripheral vasodilation that is observed in the pregnant women (Lopez-Jaramillo, 1996). Nowadays there is evidence that demonstrate that this peripheral vasodilation is product of an increase in the production of NO and prostacyclin in vascular endothelial cells (Lopez-Jaramillo et al., 2008a; Félix et al., 1991). In addition, these substances suppress the leukocyte and platelet migration and adhesion to the vascular wall (Moncada et al., 1991). The NO synthases (NOSs) are a family of specialized enzymes that synthesize NO from the amino acid L-arginine. The endothelial NOS (eNOS) depends on NADPH and calcium (Moncada et al., 1991), and we have proven the critical role of concentrations of extracellular calcium in the production of endothelial NO and in the control of vascular tone via the activation of cGMP (Lopez-Jaramillo et al., 1990a) Importantly, eNOS is expressed in the human placental syncytiotrophoblasts and in the extravillous trophoblasts, suggesting that the production of NO in the placenta play an important role in the vascular adaptations that are necessary to guarantee a normal blood flow (Sladek et al., 1997). The reports showing that NO is inactivated by superoxide (O^-2^) and that this free radical is increased in pregnancies complicated with subclinical infections or low degree inflammation, suggest that these risk factors for preeclampsia are associated to a lower activity of NO in response to an increase oxidative stress (Lopez-Jaramillo et al., 2008a). In reality, it is now well-accepted than the production of NO in the vascular endothelium of all vascular beds play an important role in the hemodynamic adaptations that are necessary to carry on a healthy pregnancy. On the other hand, a lower production and/or an increased inactivation of NO explain the generalized peripheral vasoconstriction, one of the most important characteristics of preeclampsia (Lopez-Jaramillo et al., 2008a). However, one of the problems to determinate the role of NO in pregnancy has been the methodological approaches to quantify the production and activity of NO. We and others groups have used the measurements of NO_2_/NO_3_ levels in plasma and serum of a small group of women with preeclampsia and compared them with the levels observed in unpregnant women and/or in women with healthy. These studies have used different methods to quantify NO_2_/NO_3_, and they have report elevated, decreased, or unchanged levels of NO_2_/NO_3_ in preeclamptic women in relation to controls (Lopez-Jaramillo et al., 2008a). Others reports measuring the levels of cGMP in plasma, urine, or platelets, demonstrated consistently that women with preeclampsia have lower levels of this mediator of the action of NO (Teran et al., 2004; Baksu et al., 2005; Lopez-Jaramillo et al., 2008a). A global analysis of these results suggests that some patients with preeclampsia have a decreased production of NO demonstrated by low levels of NO_2_/NO_3_ and cGMP, but in other cases there is a normal or increased production of NO, as demonstrated by the presence of similar or higher levels of NO_2_/NO_3_ in relation to women with healthy pregnancies, but in these cases they also presented with a decreased bioactivity of NO, demonstrated by the low levels of cGMP (Lopez-Jaramillo et al., 1996). Importantly, to support the role of NO in pregnancy, there are the consistent results reporting that low mediated dilation in women with healthy pregnancies have a higher vasodilation response to hyperemia that the one observed in women with preeclampsia, even before clinical manifestations appeared (Lopez-Jaramillo et al., 2008a). The analysis of the different reports of the L-arginine-NO pathway led us to the conclusion that in face of the multicausality of this disease, there are different mechanisms that can explain the alterations in the activity of the NO (Lopez-Jaramillo, 2000; Lopez-Jaramillo et al., 2001b). For instance, we understand that in the cases of preeclampsia with low NO production (low NO_2_/NO_3_ levels) the alterations are related with a deficiency in the substrate L-arginine, a deficit in the cofactors that are needed for a normal activity of the eNOS, such as ionic calcium and BH_4_, build-up of the endogenous inhibitor of eNOS, the asymmetric dimethylarginine (ADMA), or by the presence of polymorphic alterations of the eNOS that result in a lower enzymatic activity (Serrano et al., 2004; Lopez-Jaramillo et al., 2008a). In the cases of preeclampsia that courses with normal or high production of NO but with a decreased NO bioactivity, there exists an increased inactivation of NO by an increased oxidative stress (Lopez-Jaramillo et al., 2008a) associated with the presence of antibodies antireceptors AT1 of angiotensin II, early abnormal placentation with placental ischemia and hypoxia, insulin resistance, subclinical infections, and overweight and obesity (Herrera et al., 2001; Herrera et al., 2007; Lopez-Jaramillo et al., 2008a). In any case, both alterations of NO explain the endothelial dysfunction observed in preeclampsia.

The presence of overweight and obesity before pregnancy, and an excessive weight gain during gestation, are causes of endothelial dysfunction and preeclampsia as demonstrated by Pardo et al reported that women with a gestational weight gain superior to 0.42 kg per week, had markers of a reduced eNOS activity and vasorelaxation in the rings of isolated umbilical veins (Pardo et al., 2015).

### Role of Endothelin in the Pathophysiology of Preeclampsia

Other studies have proposed a role for endothelin (ET-1) in preeclampsia. ET-1 is a 21 amino acid active oligopeptide derived from a bigger precursor known as preproendothelin. This active peptide is a powerful vasoconstrictor that acts binding to the endothelin type A (ET_A_) receptor, located in vascular smooth muscle cells. Increased levels of ET-1 have been reported in women with preeclampsia, compared to the levels observed in a healthy pregnancy (Rust et al., 1997). Moreover, it looks like there is a correlation between the levels of ET-1 and the severity of the disease (Nova et al., 1991). It has been proposed that the reduced NO production that occurs in the placenta in conditions of hypoxia increases the production of ET-1. Some experimental evidences support this proposal, for instance it was reported that the administration of L-arginine reduces the levels of renal cortical preproET-1 mRNA expression in pregnant rats infused with sFlt-1 (Spradley et al., 2015). This result supports the view that NO regulates the synthesis of ET-1 in the kidney, and that when the production NO is decreased by an ischemic placenta, the synthesis of ET-1 increases with the consequent vasoconstrictor and hypertensive effects. Moreover, it has been reported that overweight and obese people with endothelial dysfunction have enhanced levels of ET-1 (Weil et al., 2011), and that an ET_A_ receptor antagonist attenuated the hypertension observed in a experimental model of rats with obesity induced by a high fat diet, that also had increased leptin levels (da Silva et al., 2004). However, in the review of Spradley et al. (2015) they found only indirect evidence that ET-1 is higher in obese preeclamptic women.

### Genetic and Epigenetic Factors Associated to Obesity and Preeclampsia

Obesity and preeclampsia are diseases that result from multiple genetic and environmental factors (Spradley et al., 2015). These two conditions share many pathophysiological mechanisms, however, only 10% of obese women will develop preeclampsia (Roberts J.M. et al., 2011). As reviewed above, the women that develop preeclampsia have different metabolic alterations that precede the appearance of clinical symptoms and that are associated with lifestyles and socio-economic status (Lopez-Jaramillo et al., 2005). We have proposed that the participation of the well-known risk factors for preeclampsia is different depending of the ethnicity and the socio-economic conditions of a specific community. The socio-economic status of communities and of individuals is determining t nutritional habits and the type and quality of the health system (World Health Organization [WHO], 1988; Lopez-Jaramillo et al., 2008a).

In the last decades, the population from low and medium incomes countries is experimenting important lifestyle changes as a result of a rapid and messy urbanization characterized by an increase in the intake of processed carbohydrates and a reduction of physical activity, conditions that have increased the prevalence of obesity and insulin resistance (Lopez-Jaramillo et al., 2001a). Moreover, ethnic differences have been reported in the prevalence of insulin resistance and metabolic syndrome (Ford et al., 2002), differences that could be associated to genetic factors or socio-economic and environmental conditions. Regardless of the cause, the relationship between insulin resistance and preeclampsia is stronger in low and medium income countries. To support this proposal the Prospective Urban and Rural Epidemiology (PURE) study have demonstrated a higher prevalence of diabetes mellitus type 2 in people with lower BMI coming from low and medium income countries compared to the prevalence found in people from high-income countries (Dagenais et al., 2016).

The existence of a role of genetic factors is demonstrated by the observation that despite of the improvement of the environmental and socio-economic factors, there is still a percentage of women that develop preeclampsia. Moreover, there is evidence showing an association between preeclampsia and polymorphisms or mutations of genes related to hypertension (Ward et al., 1993; Grandone et al., 1997; Sohda et al., 1997; Kupferminc et al., 1999; Ford et al., 2002). However, it looks like environmental and socio-economic factors have a protagonic role. For instance, various studies originated in Europe have demonstrated that pregnant women with preeclampsia have increased levels of ADMA, which are associated with endothelial dysfunction (Fickling et al., 1993; Holden et al., 1998; Ellis et al., 2001; Savvidou et al., 2003). Early in our research, we showed no difference in ADMA plasma levels between a small sample of Andean women: 22 unpregnant women, 22 women with a normal pregnancy and 22 pre-eclmaptic women (Lopez-Jaramillo et al., 1996). This result was later confirmed in a study (Maas et al., 2004) that included a bigger sample of Colombian women (67 women with preeclampsia and 93 healthy controls). We have proposed some possible explanations for this unexpected finding, to finally conclude that the probable reason is that the excess of the other risk factors present in our population is more important than ADMA plasma levels in the development of preeclampsia, situation that differs from that observed in the European population, where risk factors such as nutritional deficiencies do not exist and others as subclinical infections are detected and treated early in pregnancy. In this way of reasoning it is possible that endothelial dysfunction is mainly related to nutritional deficiencies, subclinical infections, and metabolic disorders in women with preeclampsia from developing countries, while genetic and immunological alterations seem to be the principal determinants for the development of preeclampsia in developed countries (López-Jaramillo et al., 2006).

**FIGURE 1:**
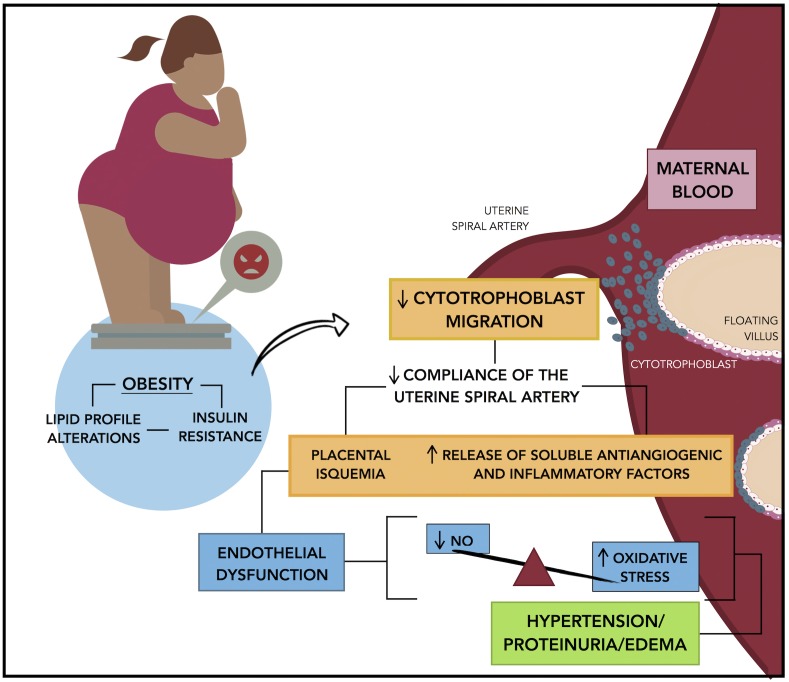
Mechanisms linking obesity to preeclampsia. Insulin resistance that results of pre-pregnancy obesity or by an excessive weight gain during gestation is associated with a reduced cytotrophoblast migration and uterine spiral artery remodeling, which in turn conduce to placental hypoxia and ischemia. In this condition the placenta release of soluble anti-angiogenic factors and inflammatory factors into the maternal circulation promoting the endothelial dysfunction, which is characterized by a decrease in the endothelial production of nitric oxide and an increase in the oxidative stress, that results in the characteristic symptoms of preeclampsia: hypertension, proteinuria, and edema.

The Figure 1 resumes the proposed mechanisms that we believe are participating in the development of preeclampsia. The evidence reviewed in this article demonstrate that the process initiate with placental alterations. A decline in cytotrophoblast migration and remodeling of the uterine spiral artery conduce to placental ischemia. In this condition, soluble anti-angiogenic and inflammatory factors are released from the placenta into the maternal circulation, these factors affect the endothelial function, a critical event that conduce to the clinical manifestations of preeclampsia.

## Conclusion

Since in medium and low-income countries the prevalence of obesity is increasing, and by the fact that preeclampsia is a major cause of maternal and perinatal morbidity and mortality in these countries, it is of crucial importance to understand how obesity impacts the pathogenesis of preeclampsia.

## Author Contributions

PL-J reviewed and approved the final version. All the authors have made a substantial contribution to this manuscript and participated in the literature review, design and redaction of the manuscript.

## Conflict of Interest Statement

The authors declare that the research was conducted in the absence of any commercial or financial relationships that could be construed as a potential conflict of interest.
